# Strategic monitoring improves prospective memory: A meta-analysis

**DOI:** 10.1177/17470218231161015

**Published:** 2023-04-03

**Authors:** Phil Peper, B Hunter Ball

**Affiliations:** 1 Department of Psychology, The University of Texas at Arlington, Arlington, TX, USA

**Keywords:** Prospective memory, strategic monitoring, attention, memory, context, meta-analysis

## Abstract

Monitoring the environment for target events that trigger prospective memory (PM) retrieval requires cognitive resources, reflected by costs to ongoing task performance (i.e., worse accuracy and/or slower response times). Strategic monitoring refers to the use of context to engage or disengage monitoring when a PM target is anticipated or unanticipated. Laboratory strategic monitoring studies have found mixed results as to whether context specification improves PM performance. This study employed a meta-analytic technique to assess the overall effect of context specification on PM performance and ongoing task metrics of strategic monitoring. Overall, context specification improved PM performance when the target was anticipated and improved ongoing task performance (speed and accuracy) when the target was not anticipated. Moderator analyses revealed the degree of slowing in anticipated contexts predicted how much context specification improved PM performance. However, the benefits to PM performance from context specification differed by the type of procedure used. PM performance was improved when context changes could be predicted during blocked or proximity procedures, but not when context varied randomly in trial-level procedures. These results provide insights into the mechanisms underlying strategic monitoring and guidance for researchers on which procedures to be use depending on the theory-driven questions.

Contextual information refers to any internal state and external environment in which an event occurs and is inextricably bound to memory. As such, context plays a pivotal role in our everyday lives and is central to numerous theoretical accounts of memory (Estes, 1955; Howard et al., 2006; Ranganath, 2010) and attention (Brosowsky & Crump, 2016; Chun, 2000). Central to the current study, the formation of prospective memory (PM) intentions—action plans to be completed in the future—involves the mental simulation of the future contexts in which the actions are to be completed (Brewer & Marsh, 2010). For example, forming the intention to pick up medication after work may include consideration of the business plaza of the pharmacy (i.e., spatial and perceptual contexts), the time in which one’s shift is over (i.e., temporal context), the frustration of driving in traffic after a long day’s work (i.e., emotional context), and the need to slow down to not miss the sharp turn into the plaza (i.e., sensorimotor context). These contextual features get bound together in a memory trace with the associated action plan and can be reactivated to facilitate intention fulfilment (Koriat et al., 1990).

Although context plays a critical role in everyday prospective remembering, most laboratory PM tasks are highly constrained and limit the use of contextual information to facilitate remembering. While controlled laboratory settings are necessary for theoretical development by limiting idiosyncratic confounds to ensure that manipulations influence the processes that are under scrutiny, this experimental control may limit the use of processes relevant for everyday remembering. This is nontrivial, as many studies have found that laboratory assessments of PM do not correlate with naturalistic PM failures (e.g., Rendell & Thomson, 1999; Schnitzspahn et al., 2011; Unsworth et al., 2012). Studying the role of context in laboratory settings can provide a better understanding of the processes underlying intention fulfilment. This study employs a meta-analytic technique to quantify how contextual information is used to strategically allocate attention resources to facilitate event-based PM. Specifically, we compare effects sizes across different laboratory paradigms and examine whether the efficacy of strategic monitoring moderates contextual benefits to intention fulfilment. By more closely aligning laboratory research with everyday remembering, we can better understand the theoretical mechanisms of PM and make practical recommendations for future researchers depending on their goals.

Most laboratory studies have investigated event-based PM, which refers to the processes by which future intentions are fulfilled in response to environmental events (i.e., PM targets), such as noticing the pharmacy and remembering to pick up medication after work. PM intentions can also be time-based or activity-based, which involve fulfilling an intention at a certain *time* (e.g., at 3 p.m.) or after a specific *activity* (e.g., after crafting an email) in the future. However, as relatively few studies have examined the role of context in these tasks, the primary focus of the current study is on event-based PM.^1^ Event-based PM intentions are first encoded (i.e., intention formation), stored in long-term memory, and retrieved from memory upon noticing the appropriate target event. PM retrieval often occurs while engaged in an ongoing task (e.g., driving), either spontaneously (i.e., automatically; McDaniel & Einstein, 2000) or through actively monitoring the environment for a target that requires additional processing to notice (R. E. Smith, 2003). Because monitoring the environment for PM targets can tax cognitive resources, it would be optimal to monitor only in contexts when the PM target (e.g., pharmacy) is anticipated. For example, monitoring can be increased near business complexes where the pharmacy is likely to occur (relevant context) and reduced in residential areas where the pharmacy is unlikely to occur (irrelevant context). In this example, location is considered the spatial context that defines the likelihood that a PM target will appear. We use the term *strategic monitoring* to describe the process of using contextual information (e.g., location) to flexibly increase monitoring when contextually appropriate (e.g., business plaza) and decrease monitoring when contextually inappropriate (e.g., Ball & Bugg, 2018a).

These real-world phenomena are typically assessed in laboratory settings by having participants form an intention to make a special PM response (e.g., press the 7 key) when they see a PM target (e.g., the *tor* syllable) embedded in an ongoing task (e.g., making lexical decisions). The primary dependent variable is PM performance, which refers to the proportion of PM targets that receive a PM response (i.e., intention fulfilment). A secondary measure typically assessed during PM tasks is ongoing task performance, which refers to the speed and accuracy of ongoing task responding. A common finding is that when the ongoing task does not orient attention to relevant features of the PM target (referred to as a nonfocal intention), monitoring the environment for PM targets produces slower and/or less accurate responding than when the same task is performed without an intention (Einstein et al., 2005). The difference in ongoing task performance between the two conditions is referred to as *cost.* Ongoing task costs are assumed to reflect allocating limited cognitive resources to monitoring for PM targets, leaving fewer for ongoing task processing that results in slower or less accurate responses (R. E. Smith, 2003; but see Strickland et al., 2018 for an alternative account). Previous research has shown monitoring costs are positively correlated with PM target detection (e.g., Ball & Brewer, 2018).

*Strategic monitoring* is typically assessed using a nonfocal PM task that encourages monitoring by instructing participants that PM targets will only occur in one contextual dimension of the ongoing task (e.g., block type, word type, location, colour, etc.). For example, participants in a *specific condition* may be (validly) instructed that PM targets will occur in Block 1 of the ongoing lexical decision task (relevant context) but not Block 2 (irrelevant context; Ball et al., 2015), or in word trials (relevant context) but not nonword trials (irrelevant context; Lourenco et al., 2013). In contrast, those in a *nonspecific condition* are (invalidly) instructed that PM targets can occur in both Block 1 (irrelevant context) and Block 2 (relevant context), or in both words (relevant context) and nonwords (irrelevant context). To be clear, participants in the nonspecific condition assume targets can occur in *any* context, so using the term “irrelevant context” is as a bit of a misnomer since all contexts are psychologically relevant to these participants. We use the terms “relevant” and “irrelevant” in reference to the instructions given to the specific condition to make easy verbal and analytical comparisons between the two conditions. Using the nonspecific condition as a comparative benchmark and analysing ongoing task performance separately for each context, results almost invariably show that costs are reduced in irrelevant contexts for the specific condition (e.g., Ball & Bugg, 2018a, 2018b; Ball et al., 2015; Cohen et al., 2012; Kuhlmann & Rummel, 2014; Lourenco et al., 2013; Lourenco & Maylor, 2014; Marsh et al., 2006; see S. M. Smith & Handy, 2016 for a review). However, results are mixed as to whether participants in the specific condition show increased costs in the relevant context or improved PM performance compared with the nonspecific condition (e.g., Ball et al., 2015; Bowden et al., 2017, 2021). The current meta-analysis aims to clarify whether increased monitoring in relevant contexts is necessary to observe PM performance benefits.

The two-process model of PM (Guynn, 2003) describes two processes involved in strategic monitoring: a prospective *retrieval mode* and *target checking*. Borrowed from the retrospective memory literature, the retrieval mode remains active throughout the entire PM task and involves maintaining a state of readiness to treat incoming stimuli as possible cues for PM intention retrieval. Target checking is an intermittent process that assesses an item for intention-related features (i.e., checking the environment for targets). Ball and Bugg (2018b) further specify that strategic monitoring requires an additional step, referred to as *context identification*, which is a process that determines whether the context is relevant or irrelevant for target checking. It is possible that this additional cognitive operation creates greater attentional demands. A task requiring participants to identify the context more frequently or unpredictably may decrease performance. Contextual manipulations used across different paradigm types place different demands on strategic monitoring processes, which may explain why sometimes PM improvements are seen and sometimes they are not.

## Primary strategic monitoring procedures

**Figure 1.**
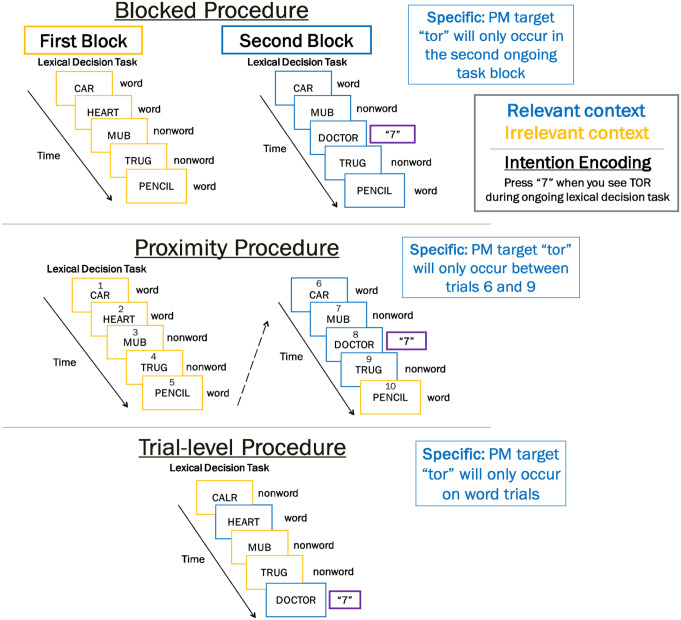
Visual depiction of different strategic monitoring procedures. *Note*. This figure displays three procedures commonly used to assess strategic PM monitoring. In the “specific” conditions, participants are instructed that PM targets (e.g., TOR syllable) will occur in a specific context (e.g., second block). This means that participants should increase monitoring in the relevant contexts (depicted in blue) and decrease monitoring in irrelevant contexts (depicted in yellow).

Strategic monitoring has largely been investigated using three different paradigms (for reviews, see Bowden et al., 2021; R. E. Smith, 2017; R. E. Smith & Skinner, 2019). We refer to these procedures as blocked, proximity, and trial-level procedures. Below, we briefly describe the methodology for each procedure (see Figure 1) and the general results found in each.

### Blocked procedure

In a blocked procedure, participants complete the task in blocks with PM targets only appearing in select blocks (i.e., relevant context; see Figure 1). This is the procedure used by Ball et al. (2015) described previously (for a similar procedure, see Meier et al., 2006). A variant of the blocked procedure includes when the block switches every few trials. For example, Bowden et al. (2021) blocked font colour in sets of four trials, with the relevant context always being in a specific colour. Results showed the specific conditions had higher PM performance and increased cost in relevant contexts than the nonspecific conditions. Experiments using a blocked procedure have shown both that context specification benefits PM performance (e.g., Ball et al., 2015) and null effects (e.g., Lourenco & Maylor, 2014). Critically, context identification in the blocked procedure happens relatively infrequently and occurs on only the first trial of (or just before) each block. The context switches also occur regularly, so participants can anticipate when the context will change. Together, these factors suggest a relatively low cognitive demand of context identification and allow participants to strategically monitor more effectively.

### Proximity procedure

The proximity procedure allows participants to track their progress towards the relevant context (see Figure 1). For example, Bowden et al. (2017) explicitly numbered each ongoing task trial and instructed participants in the specific condition that PM targets would only appear in a certain window of trials (e.g., 25–30, 45–50, 70–75, and 95–100). They found that context specification improved PM performance, increased monitoring costs in the relevant context, and decreased costs in the irrelevant context. The proximity procedure has also been implemented using a familiar environment (R. E. Smith et al., 2017) and colours (Bowden et al., 2021; Experiment 1) to provide information about the approaching relevant context. PM performance was improved significantly in Bowden et al. (2021), but only marginally in R. E. Smith et al. (2017). Critically, context identification in the proximity procedure happens relatively infrequently and the context information allows participants to anticipate when the context will change. Together, these factors suggest a relatively low cognitive demand of context identification and allow participants to strategically monitor more effectively.

### Trial-level procedure

The trial-level procedure assigns a relevant context to a set of ongoing task stimuli that varies randomly on every trial (Figure 1). For example, Lourenco et al. (2013) instructed participants to look for a specific syllable (e.g., *tor*) in an ongoing lexical decision task. Participants in the specific condition were instructed that PM targets would appear on word trials (i.e., relevant context) but not nonword trials (i.e., irrelevant context), whereas those in the nonspecific condition thought targets could occur in any trial. Participants in the specific condition showed decreased monitoring costs in irrelevant contexts compared with participants in the nonspecific condition, but there was no difference in PM performance or costs in the relevant context. Other studies using the trial-level procedure have manipulated context with colour (Lourenco & Maylor, 2014), location (Bugg & Ball, 2017), and shapes (Kuhlmann & Rummel, 2014). Some studies have found PM performance benefits (e.g., Loft et al., 2011b), while many others have not (e.g., Ball & Bugg, 2018b). Critically, context identification is required on every trial in the trial-level procedure as context switches occur randomly. Participants therefore cannot anticipate when the context will change. Together, these factors suggest a comparatively high cognitive demand of context identification.

## Current study

This study aimed to distinguish and quantify factors at the meta-analytic level that account for the finding that sometimes context specification improves PM performance and other times it does not. Experiments were coded for strategic monitoring procedure and labelled either blocked, proximity, or trial-level. The effect of context specification on PM performance was the primary dependent variable of interest. We were also interested in the possible moderating role of strategic monitoring cost metrics. Overall, we predicted that context specification would benefit PM performance—that is, participants in specific conditions would show better PM performance than those in nonspecific conditions. We also predicted overall that context specification would increase monitoring cost (i.e., slower RTs and worse ongoing task accuracy) in relevant contexts and decrease monitoring cost (i.e., faster RTs and better ongoing task accuracy) in irrelevant contexts due to strategic monitoring. However, we predicted that the extent context specification benefitted PM performance would differ by procedure type and by the amount of strategic monitoring exhibited in the relevant context. Specifically, we predicted context specification would improve PM performance in the blocked and proximity procedures, but not in the trial-level procedure. We also predicted that greater strategic monitoring in the relevant context (i.e., slower RTs and/or worse ongoing task accuracy in the specific condition) would enlarge the effect of context specification on PM performance. However, we predicted no relationship between PM performance and strategic monitoring in the irrelevant context (i.e., faster RTs and/or better ongoing task accuracy in the specific condition).

The primary motivating hypothesis was that the three strategic monitoring procedures place differing demands on the processes underlying strategic monitoring. Specifically, context identification varies substantially between procedures. The difference can most clearly be seen in the blocked versus trial-level procedures. In the blocked procedure, context identification occurs at the beginning of each block (or the first trial), whereas the trial-level procedure requires context identification on *every trial*. Lourenco and Maylor (2014) compared performance on trial-level versus blocked (sets of eight trials) procedures. When context was blocked, strategic monitoring costs were larger than when context varied trial-by-trial, suggesting that engaging and disengaging monitoring is more effective when context identification is easier. Kuhlmann and Rummel (2014) have noted that context identification is essentially another PM intention, such that one must remember to check the context on every trial before deciding to target check or not (i.e., engaging or disengaging monitoring). The greater demands on every relevant context trial in trial-level procedures may lead to the typical null effect of context specification on PM performance. This idea is supported by the findings of Bowden et al. (2021) who provided pretrial warnings of the upcoming relevant context in a blocked procedure (i.e., sets of four relevant context trials). They found that a pretrial warning was essential for context specification to benefit PM performance on targets in the *first* trial of the relevant context. However, targets appearing in the other relevant context trials showed a PM performance benefit regardless of the pretrial warning, because the relevant context was already identified on the first trial. This suggests that context identification must *precede* a relevant context trial for context specification to benefit PM performance on that trial. Consistent with the robust monitoring costs and benefit to PM performance observed in Bowden et al. (2017), the proximity procedure lessens demands on context identification, as participants can track their exact progress in relation to relevant context and predict a relevant context trial prior to trial onset. Given the varied demands on context identification, we predicted context specification would benefit PM performance more in the blocked and proximity procedures compared with the trial-level procedure.

## Methods

This study followed the guidelines for Preferred Reporting Items for Systematic Reviews and Meta-Analyses (PRISMA). It was preregistered on Open Science Framework and data are publicly available at https://osf.io/hps7g.

### Study selection

The databases that were searched include PubMed, Google Scholar, PsycINFO, and PsyArxiv (preprints). The search terms selected are *prospective memory, delayed intentions, context, strategic monitoring, preparatory attention, costs*, and *task interference*. Search terms were selected based on the keywords found in six related papers ranging from 2006 to 2021 to cover a range of historical terms describing the topic. The references of book chapters and an article with an introduction that qualitatively reviewed existing strategic monitoring papers were scanned (Bowden et al., 2021; R. E. Smith, 2017; R. E. Smith & Skinner, 2019). The stopping date for the literature search was July 23, 2021. Each article was selected and coded by the first author. Ambiguities were clarified by second author who has published multiple papers on the topic.

#### Inclusion criteria

**Figure 2.**
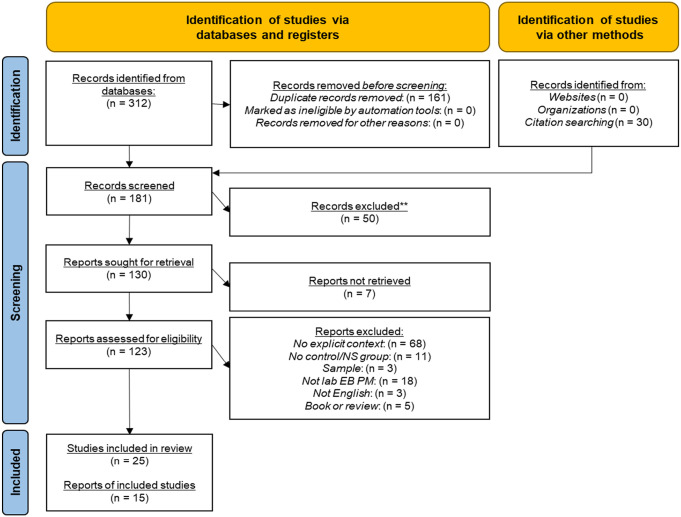
PRISMA flow diagram depicting steps of the database search and exclusion process.

To be included in the analyses, the study met the following criteria (see Figure 2 for PRISMA flow diagram (Page et al., 2021) depicting study search and selection):

1. Only studies with data from healthy younger adult samples with ages between 18 and 35 years. Although strategic monitoring has been observed in older adults (Ball & Bugg, 2018a), that population may not be able to utilise contextual information as effectively as younger adults (Ball et al., 2020).
2. The quantitative experimental or observational data came from either a peer-reviewed publication, preprint, or dissertation/thesis published between 1990 and 20 June 2021.
3. One or more laboratory event-based PM tasks manipulating context in the study were required, such that at least one condition was aware of the PM context (i.e., specific) and one condition was unaware (i.e., nonspecific) of the PM context. The PM task must have included more than one PM trial, as there are reliability concerns in tasks with only a single PM trial (Maylor, 1993, 1996). In addition, only PM tasks with nonfocal intentions (not focal intentions) were included because monitoring is not required when the intention is focal (Einstein & McDaniel, 2005).
4. The article was written in English.

#### Exclusionary criteria

1. Qualitative or naturalistic data and systematic reviews were excluded from analysis. Data from book chapters were excluded to avoid redundancy with data reported from articles (see Kliegel et al., 2008 for a similar procedure).
2. Data from studies with samples using children (<18 years old), middle and older adults (36 years and older), clinical populations with psychopathology that might influence results (e.g., schizophrenia), traumatic brain injury, and early-onset dementia were not included in the analysis.

### Dependent variables

#### PM performance

The proportion of PM targets detected in the relevant context.

#### Ongoing task response times

Mean response times in the PM block on nontarget trials in the relevant and irrelevant contexts.

#### Ongoing task accuracy

Mean accuracy in the PM block on nontarget trials in the relevant and irrelevant contexts.

#### Ongoing task costs

Costs were calculated by subtracting ongoing task performance (accuracy and response times) in a control block or control group’s (no intention) from the performance in a PM block (with a PM intention). Note that cost analyses produced nearly identical results to standard RT and accuracy measures. However, fewer studies had control blocks to calculate cost scores, so we did not report these effects in the paper and instead only reported pure accuracy and response times that also reflect monitoring.

### Subgroup and moderator analysis

After all the relevant papers were identified, the first author coded each experiment for the subgroups based on procedure type. In addition, monitoring (response times and accuracy) differences between specific and nonspecific conditions in both relevant and irrelevant contexts will be included as moderators.

#### Subgroups

Different subgroups were coded by the type of strategic monitoring procedure used in each experiment. The three procedures described previously were blocked, proximity, and trial-level. Blocked procedures were identified by PM trials appearing in a subset of multiple task blocks. That is, relevant and irrelevant contexts were grouped in blocks of trials (e.g., Ball et al., 2015). Proximity was identified by task parameters (e.g., numbered trials and spatial location) that allowed participants to track progress and relative position in relation to an upcoming relevant context (e.g., Bowden et al., 2017). Trial-level procedures were identified by both relevant and irrelevant context trials that appeared within the same block and presented randomly (e.g., Lourenco et al., 2013). Fu et al. (2011) recommended a minimum of four studies in each group of a subgroup analysis.

#### Moderators

The effect size for differences between the specific condition and the nonspecific condition for ongoing task response times, accuracy, and cost served as the moderators. Moderator analyses with cost scores mirrored the patterns observed for moderator analyses for response times and accuracy, only with fewer studies. Only the latter moderator analyses were reported.

### Analyses

The programme Meta-Essentials was used to conduct the analyses (Suurmond et al., 2017). Effect sizes for comparisons between mean proportion of PM performance and ongoing task performance in the relevant and irrelevant contexts were calculated with Hedge’s *g*. Means and standard deviations for all outcome variables, separated by specific and nonspecific conditions, were extracted from included articles or obtained by authors when unavailable. Some data were not relevant (e.g., ongoing task accuracy in Loft et al., 2011a) for analysis. Each exclusion and the reason are described below.

Ongoing task response data in the relevant context was applicable in 25 (response times) and 24 (accuracy) out of the 25 total studies. Loft et al. (2011) used an air-traffic control version of an event-based PM in which the equivalent of the ongoing task had a single response. That is, there was no correct response to record accuracy. However, Loft et al. argue that longer response times still reflect target checking behaviour, so their response times were included in our analyses. Ongoing task response data in the irrelevant context was applicable in 24 (response times) and 23 (accuracy) out of the 25 total studies. In Brewer and Marsh (2010), participants in the nonspecific condition formed an intention to make a PM response to any animal at the beginning of the experiment. The specific condition was informed that the animal words would appear only in a lexical decision task (i.e., relevant context). That is, the entire lexical decision task was the relevant context and irrelevant context consisted of the procedure outside of the lexical decision task. Therefore, there was no useable ongoing task data in the irrelevant context for this experiment. Finally, an important note should be made about how relevant and irrelevant contexts were defined in R. E. Smith et al. (2017). In those two experiments, participants were undergraduate students who completed an ongoing task that involved viewing pictures of people in various locations around the university the participants all attended. Participants were to make judgements about whether there were six or more people in the picture. The PM intention was to make a PM response at specified locations around the university. The specific condition received the ongoing task pictures organised in a spatial order (i.e., mimicking a walk around campus) that allowed them to predict an upcoming PM target, whereas the pictures were presented randomly in the nonspecific condition. In all other strategic monitoring experiments, the relevant context boundaries were clearly demarcated (e.g., second block of lexical decisions, trials 25–30, word trials, etc.). R. E. Smith et al. (2017) instead had “soft” boundaries separating contexts. In their analyses, Smith et al. binned ongoing task performance into three subintervals varying in distance to the PM target. That is, the first subinterval represented trial locations farthest from the PM target and the third subinterval represented locations nearest the PM target. For the analyses in the current meta-analysis, we coded the irrelevant context in R. E. Smith et al. (2017) as the mean ongoing task performance of subintervals one and two and used subinterval three ongoing task performance as the relevant context.

In each of analysis, we reported *I*^2^, *Q*, and *T*^2^ as indices of heterogeneity, though we used *I*^2^ for interpretation (see Leong et al., 2019). Higgins et al. (2003) interprets an *I*^2^ of 25% as low, 50% as moderate, and 75% as high. Moderate heterogeneity (> 50%) can warrant further analyses (subgroup and moderator) to explain the range of effects (Harrer et al., 2019). Prediction intervals outline the range of observed effect sizes in the meta-analysis. Prediction intervals spanning zero mean that future studies may observe null effects even when a true difference exists (Hak et al., 2016). All analyses used random effects models to account for the heterogeneity across studies using inverse variance weighting (i.e., large confidence intervals were given less weight). Subgroup analyses were performed by averaging the weight separately for each subgroup.

Four experiments used within-subjects design for the specific and nonspecific conditions. Because of differences in how effect sizes are calculated for between- and within-subjects differences, effect sizes for all experiments could not be directly calculated based on means and standard deviations in the same between-subjects meta-analysis. We therefore calculated effect sizes for the within-subjects design experiments separately and imputed the *Hedge’s g* calculated for each into the overall meta-analysis for each dependent variable (Morris & DeShon, 2002).

## Results

### Overall PM effects

**Figure 3.**
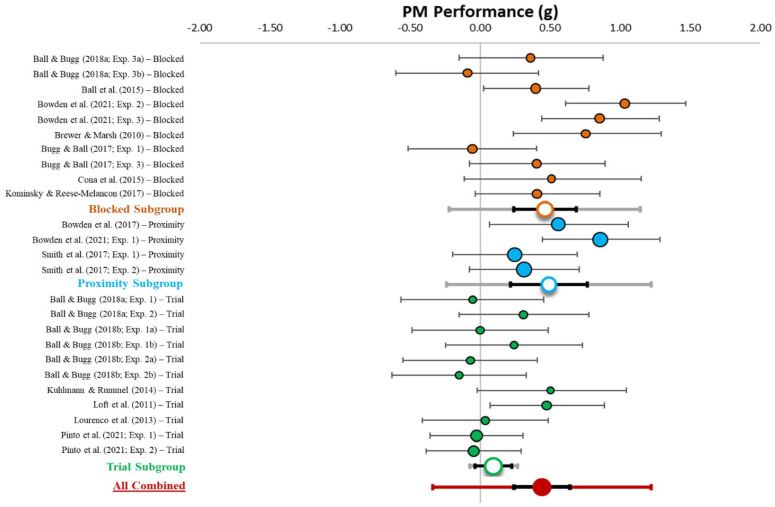
Forest plot and subgroup analysis for the effect of context specification on PM performance. *Note.* This figure shows the individual (coloured circles with black outlines), subgroup combined (white circles with coloured outlines), and overall combined (red circle) effect sizes for the effect of context specification on PM performance with the relative weight of each data point on the left side. Black error bars reflect 95% confidence intervals and grey (subgroup) and red (overall combined) error bars reflect 95% prediction intervals.

Table 1 describes effect sizes and heterogeneity indices for the differences in PM performance between specific and nonspecific conditions, which is also displayed in Figure 3. The forest plot in Figure 3 displays the distribution of weighted mean effect sizes (circles), 95% confidence intervals (CIs; black error bars), and 95% prediction interval (PI; green error bar) for the difference in PM performance between specific and nonspecific conditions. CIs reflect the range of the true effect. This analysis (*k* = 25) revealed a combined effect (red circle) that was small-to-medium in size (*g* = .31, *z* = 4.55, *p* < .001, 95% CI [0.17, 0.45], 95% PI [−0.24, 0.85]). Critically, the confidence interval does not overlap with zero, suggesting that having context information about when a PM target will appear improved the likelihood of detecting that target. However, the prediction interval spanned zero, suggesting that future studies of strategic monitoring may find null effects. Notably, however, there was moderate heterogeneity (*Q* = 55.16, *p* < .001, *I*^2^ = 56.49, *T*^2^ = .07), suggesting that subgroup and moderator analyses are needed to explain systematic variations in effect sizes.

**Table 1.** List of studies, designs, means, effect sizes, confidence and prediction intervals, heterogeneity, and moderator values for PM performance.

DV	Study	Exp	Design	Type	K	*N* (S)	*N* (NS)	Mean (S)	Mean (NS)	Hedge’s *g* (*SE*)	95% CI	95% PI	*Q*	*I* ^2^	*T* ^2^	Moderator (Hedge’s g)			
																Rel RT	Irrel RT	Rel ACC	Irrel ACC
PM performance	Ball & Bugg (2018a)	3a	Between	Blocked	—	30	30	0.93	0.87	0.36	[−0.15, 0.88]	—	—	—	—	−0.02	—1.13	0.3245	0.2081
	Ball & Bugg (2018a)	3b	Within	Blocked	—	30		0.78	0.75	−0.09	[−0.6, 0.42]	—	—	—	—	0.20	−0.43	0.19	0
	Ball et al. (2015)	1	Between	Blocked	—	56	56	0.83	0.71	0.40	[0.02, 0.78]	—	—	—	—	0.26	−0.27	0	0
	Bowden et al. (2021)	2	Between	Blocked	—	48	48	0.84	0.64	1.03	[0.61, 1.47]	—	—	—	—	0.14	−0.83	−0.4209	0.1984
	Bowden et al. (2021)	3	Between	Blocked	—	48	48	0.76	0.59	0.85	[0.44, 1.28]	—	—	—	—	0.69	−0.33	0	0.1984
	Brewer & Marsh (2010)	1	Between	Blocked	—	30	30	0.54	0.31	0.75	[0.24, 1.29]	—	—	—	—	0.29	—	0.12	—
	Bugg & Ball (2017)	1	Between	Blocked	—	37	37	0.89	0.90	−0.05	[−0.51, 0.40]	—	—	—	—	0.14	−0.53	0.4948	0.1649
	Bugg & Ball (2017)	3	Between	Blocked	—	35	33	0.86	0.75	0.40	[−0.08, 0.89]	—	—	—	—	0.01	−0.81	−0.1648	−0.1648
	Cona et al. (2015)	1	Between	Blocked	—	21	20	0.87	0.80	0.51	[−0.11, 1.15]	—	—	—	—	0.19	−0.65	−0.3077	−0.2688
	Kominsky & Reese-Melancon (2017)	1	Between	Blocked	—	40	40	0.45	0.29	0.41	[−0.04, 0.85]	—	—	—	—	0.38	0.08	0	−0.1228
	Bowden et al. (2017)	1	Between	Proximity	—	32	34	0.83	0.70	0.56	[0.07, 1.06]	—	—	—	—	0.24	−0.45	−0.0205	0.3294
	Bowden et al. (2021)	1	Between	Proximity	—	48	48	0.74	0.55	0.86	[0.44, 1.28]	—	—	—	—	0.70	−0.23	0.248	0
	R. E. Smith et al. (2017)	1	Between	Proximity	—	40	39	0.85	0.78	0.25	[−0.2, 0.69]	—	—	—	—	−0.27	−0.68	0.39	0.00
	R. E. Smith et al. (2017)	2	Between	Proximity	—	52	50	0.80	0.71	0.31	[−0.08, 0.71]	—	—	—	—	−0.36	−0.50	0.31	0.19
	Ball & Bugg (2018a)	1	Between	Trial	—	30	30	0.84	0.85	−0.05	[−0.57, 0.45]	—	—	—	—	−0.16	−0.43	0	0.436
	Ball & Bugg (2018a)	2	Between	Trial	—	37	37	0.87	0.80	0.31	[−0.15, 0.77]	—	—	—	—	−0.12	−0.49	0.48	0
	Ball & Bugg (2018b)	1a	Within	Trial	—	33		0.70	0.72	0.00	[−0.49, 0.49]	—	—	—	—	−0.21	−0.46	0	0
	Ball & Bugg (2018b)	1b	Within	Trial	—	33		0.70	0.75	0.24	[−0.24, 0.73]	—	—	—	—	0.14	0.11	0	0
	Ball & Bugg (2018b)	2a	Within	Trial	—	34		0.70	0.71	−0.07	[−0.55, 0.41]	—	—	—	—	−0.21	−0.88	0	0
	Ball & Bugg (2018b)	2b	Within	Trial	—	34		0.70	0.62	−0.15	[−0.63, 0.33]	—	—	—	—	−0.17	−0.19	0	0.21
	Kuhlmann & Rummel (2014)	1	Between	Trial	—	27	30	0.64	0.53	0.50	[−0.02, 1.04]	—	—	—	—	0.54	−0.27	0	0.2671
	Loft et al. (2011)	1	Between	Trial	—	48	48	0.88	0.79	0.47	[0.07, 0.89]	—	—	—	—	0.15	−0.58	—	—
	Lourenco et al. (2013)	1	Between	Trial	—	38	39	0.78	0.77	0.04	[−0.41, 0.49]	—	—	—	—	0.21	−0.37	0.219	0.3461
	Pinto et al. (2022)	1	Between	Trial	—	71	70	0.71	0.72	−0.03	[−0.36, 0.3]	—	—	—	—	0.03	−0.44	0	0.1405
	Pinto et al. (2022)	2	Between	Trial	—	68	67	0.83	0.84	−0.05	[−0.39, 0.29]	—	—	—	—	0.02	−0.29	−0.4318	0.1408
	Combined (all)	—		All	25	—	—	0.77	0.70	0.31 (0.07)	[0.17, 0.45]	[−0.24, 0.85]	55.16	56.49%	0.07	**—**	**—**	**—**	**—**
	Blocked subgroup	—		Blocked	10	—	—	0.78	0.66	0.46	[0.24, 0.69]	[−0.22, 1.14]	21.72	58.56%	0.08	**—**	**—**	**—**	**—**
	Proximity subgroup	—		Proximity	4	—	—	0.81	0.69	0.49	[0.21, 0.77]	[−0.24, 1.22]	5.06	40.66%	0.03	**—**	**—**	**—**	**—**
	Trial subgroup	—		Trial	11	—	—	0.76	0.74	0.10	[−0.04, 0.23]	[−0.08, 0.27]	10.28	2.72%	0.03	**—**	**—**	**—**	**—**

PM: prospective memory; DV: dependent variable; S: specific condition; NS: nonspecific condition; CI: confidence interval; PI: prediction interval; Rel RT: relevant context response time; Irrel RT: irrelevant context response time; Rel ACC: relevant context accuracy; Irrel ACC: irrelevant context accuracy.

### PM performance subgroup analysis

Subgroup analyses for procedure type were run to further explain the heterogeneity in the degree to which context specification influenced PM performance. The forest plot for the subgroup analysis is displayed in Figure 3. The white circles with coloured outlines reflect the combined weighted effect size for each subgroup (e.g., the orange circle reflects the blocked subgroup). The between group analysis of variance (ANOVA) indicated significant differences in PM performance between procedures (*p* = .003).

#### Blocked

In the blocked procedure, heterogeneity was moderate (*Q* = 21.72, *p* = .010, *I*^2^ = 58.56, *T*^2^ = .08). There was a medium-sized combined effect (*g* = 0.46, 95% CI [0.24, 0.69], [−0.22, 1.14]). Critically, the confidence intervals did not include zero, but the prediction intervals did span zero.

#### Proximity

In the proximity procedure, heterogeneity was low (*Q* = 5.06, *p* = .168, *I*^2^ = 40.66, *T*^2^ = .03). There was a medium-sized combined effect (*g* = 0.49, 95% CI [0.21, 0.77], 95% PI [−0.24, 1.22]). Critically, the confidence interval did not include zero, but the prediction interval did span zero.

#### Trial-level

In the trial-level procedure, heterogeneity was low (*Q* = 10.28, *p* < .001, *I*^2^ = 2.72, *T*^2^ = .00). There was a small-sized combined effect (*g* = 0.10, 95% CI [−0.04, 0.23], 95% PI [−0.08, 0.23]). Critically, the confidence and prediction intervals included zero.

#### Summary

Consistent with predictions, the confidence interval results indicated that context specification improved PM performance in the blocked and proximity procedures, but not the trial-level procedure.

### Moderator analysis

Moderator analyses were run to further explain the heterogeneity in the degree to which context specification influenced PM performance.

#### Relevant context

**Figure 4.**
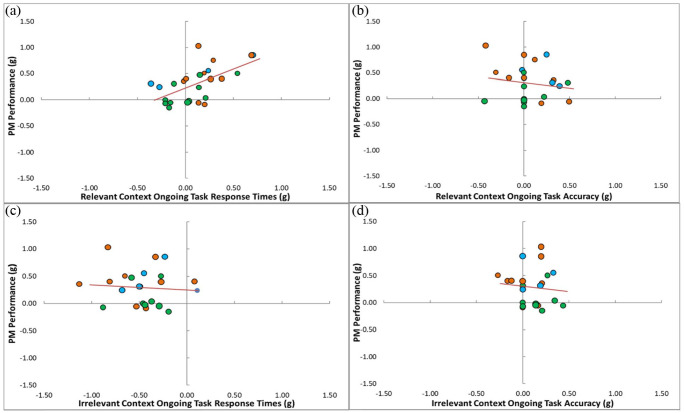
Moderator analysis for the effect of context specification on ongoing task performance as a function of the effect of context specification on PM performance. *Relevant context*: (a) reflects response times and (b) accuracy in the relevant context. *Irrelevant context*: (c) reflects response times and (d) reflects accuracy in the irrelevant context. *Note.* Ongoing task (response time and accuracy) moderator analyses on the effect of context on PM performance. Orange circles represent experiments with a blocked procedure, blue circles represent experiments with a proximity procedure, and green circles represent experiments with a trial-level procedure.

For response times (*k* = 25), positive moderator values (Figure 4a) represent larger (slower) response times in the specific condition (i.e., increased monitoring in relevant contexts with specification). Figure 4a shows that the heterogeneity observed in the effect of specification on PM performance can be explained well by the effect of specification on relevant context response times. The meta-regression moderator analysis revealed the specification benefit to PM performance was positively related to the degree to which specification slowed response times in relevant contexts (β = .60, *p* < .001). For accuracy (*k* = 24), negative moderator values (Figure 4b) represent less accurate responding in the specification condition (i.e., increased monitoring in relevant contexts with specification). The meta-regression moderator analysis revealed the effect of specification on PM performance was unrelated to the effect of specification on nontarget response accuracy in the relevant context (β = −.16, *p* = .467). Thus, PM performance was improved when context specification slowed response times in the relevant context, but this pattern was not observed for accuracy.

#### Irrelevant context

For response times (Figure 4c), negative moderator values represent smaller (faster) response times in the specific condition (i.e., reduced monitoring in irrelevant contexts with specification). The meta-regression moderator analysis (*k* = 24) revealed the effect of specification on PM performance was not significantly related to the effect of specification on response times in irrelevant contexts (β = −.08, *p* = .721). For accuracy (Figure 4d), positive moderator values represent more accurate responding in the specific condition (i.e., more accurate in irrelevant contexts with context information). The meta-regression moderator analysis (*k* = 23) revealed the effect of specification on PM performance was unrelated to the effect of specification on response accuracy on trials in the irrelevant context (β = −.10, *p* = .659). Thus, PM performance was not improved when specification was used to reduce monitoring in the irrelevant context.

#### Publication bias

**Figure 5.**
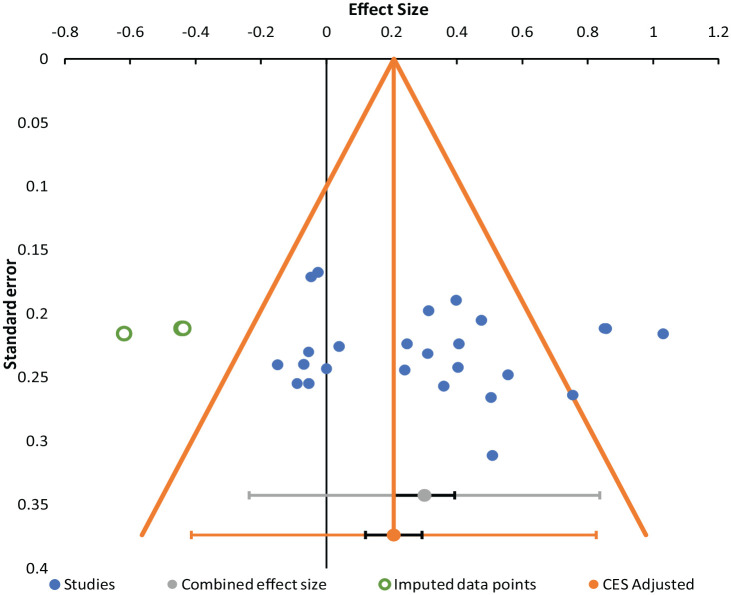
Funnel plot of studies in the prospective memory performance analysis. *Note*. The funnel plot represents effect sizes (*x*-axis) and standard error (*y*-axis) for individual studies (blue dots), imputed data points (green circles), combined effect size (grey dot), and adjusted effect size (orange dot). Confidence intervals are represented by the black bars and prediction intervals by the wider grey and orange bars for the combined and adjusted effect sizes, respectively. The adjusted effect size is lower because it is calculated with the imputed data left of the funnel.

The funnel plot in Figure 5 shows the distribution of effect sizes across all experiments for the difference in PM performance between specific and nonspecific conditions. Funnel plots are typically used as an assessment of publication bias, as studies with minimal heterogeneity and no reporting bias should vary randomly around the combined effect size (horizontal axis). Studies with more power have smaller standard errors (vertical axis), and the triangle reflects 1.96 standard error on each side to visualise whether effect sizes are randomly distributed and if certain studies fall outside of this interval. The funnel plot shows a reasonably random distribution of effect sizes with only three studies falling outside of this interval. However, due to apparent asymmetry in the effect size distribution, Meta-essentials uses a Trim-and-Fill method to impute “missing studies” with effect sizes that round out the distribution (green circles). An adjusted combined effect size is then calculated that accounts for imputed data. While the visual asymmetry in effect sizes indicate there may be publication bias, the nonsignificant Egger regression (*p* = .548) and a nonsignificant Begg and Mazumdar’s rank correlation (*p* = .709) tests for asymmetry suggest the risk of publication bias is minimal. We do not interpret the adjusted combined effect size due to the minimal risk of publication bias, though the adjusted effect size is still significant.

### Primary analysis summary

At the meta-analytic level, context specification benefitted PM performance, though the effect was small-to-medium in size. This effect differed by subgroup, with medium-sized effects observed in the blocked and proximity procedures and a nonsignificant effect observed in the trial-level procedure. Separating studies into procedural subgroups partially explained the moderate heterogeneity in effect sizes, because heterogeneity was low among studies with a trial-level and proximity procedures but remained moderate in the blocked procedure. The moderator analyses showed when specification increases monitoring (slower responding) in relevant contexts, specification tends to benefit PM performance, but using specification to reduce monitoring in irrelevant contexts does not. Response accuracy in relevant and irrelevant trials was not related to PM performance.

### Overall response time and accuracy effects

The following analyses examine how context specification influences ongoing task performance (i.e., speed and accuracy) on nontarget trials. These analyses can be seen as complementary to the above moderator analyses showing that changes in ongoing task performance during relevant and irrelevant contexts can influence PM. Subgroups were assessed for each analysis to determine whether monitoring differed across procedures. We made no predictions between procedure subgroups for ongoing task performance.

#### Relevant context response times

Table 2 describes effect sizes and heterogeneity indices for the response time differences in relevant contexts between specific and nonspecific conditions, which are also displayed in Figure 4. The meta-analysis (*k* = 25) revealed a small-sized combined effect (*g* = .11; *z* = 1.90, *p* = .057, 95% CI [−0.01, 0.22], 95% PI [−0.26, 0.48]). Critically, the confidence and prediction intervals include zero. This suggests that having information about when a PM target will appear has no overall effect on ongoing task response times when the target is relevant. There was low heterogeneity among effect sizes (*Q* = 38.11, *p* = .034; *I*^2^ = 37.03; *T*^2^ = .03).

**Table 2.** List of studies, designs, means, effect sizes, confidence and prediction intervals, and heterogeneity indices for relevant context RTs.

DV	Study	Exp	Design	Type	K	*N* (S)	*N* (NS)	Mean (S)	Mean (NS)	Hedge’s *g* (*SE*)	95% CI	95% PI	Q	*I* ^2^	*T* ^2^
Relevant ongoing task response time	Ball & Bugg (2018a)	3a	Between	Blocked	—	30	30	625.00	628.00	−0.02	[−0.53, 0.49]	—	—	—	—
	Ball & Bugg (2018a)	3b	Within	Blocked	—	30		745.00	720.00	0.20	[−0.31, 0.71]	—	—	—	—
	Ball et al. (2015)	1	Between	Blocked	—	56	56	727.00	698.00	0.26	[−0.11, 0.64]	—	—	—	—
	Bowden et al. (2021)	2	Between	Blocked	—	48	48	937.00	912.00	0.14	[−0.26, 0.54]	—	—	—	—
	Bowden et al. (2021)	3	Between	Blocked	—	48	48	1,032.00	874.00	0.69	[0.28, 1.11]	—	—	—	—
	Brewer & Marsh (2010)	1	Between	Blocked	—	30	30	832.00	792.00	0.29	[−0.22, 0.81]	—	—	—	—
	Bugg & Ball (2017)	1	Between	Blocked	—	37	37	656.00	635.00	0.14	[−0.32, 0.6]	—	—	—	—
	Bugg & Ball (2017)	3	Between	Blocked	—	35	33	665.00	664.00	0.01	[−0.47, 0.49]	—	—	—	—
	Cona et al. (2015)	1	Between	Blocked	—	21	20	915.00	883.00	0.19	[−0.42, 0.82]	—	—	—	—
	Kominsky & Reese-Melancon (2017)	1	Between	Blocked	—	40	40	713.00	667.00	0.38	[−0.06, 0.83]	—	—	—	—
	Bowden et al. (2017)	1	Between	Proximity	—	32	34	1,958.00	1,843.00	0.24	[−0.25, 0.73]	—	—	—	—
	Bowden et al. (2021)	1	Between	Proximity	—	48	48	1,031.00	848.00	0.70	[0.29, 1.12]	—	—	—	—
	R. E. Smith et al. (2017)	1	Between	Proximity	—	40	39	1,725.00	1,884.00	−0.27	[−0.72, 0.17]	—	—	—	—
	R. E. Smith et al. (2017)	2	Between	Proximity	—	52	50	1,273.00	1,454.00	−0.36	[−0.75, 0.03]	—	—	—	—
	Ball & Bugg (2018a)	1	Between	Trial	—	30	30	878.00	897.00	−0.16	[−0.67, 0.35]	—	—	—	—
	Ball & Bugg (2018a)	2	Between	Trial	—	37	37	849.00	866.00	−0.12	[−0.58, 0.34]	—	—	—	—
	Ball & Bugg (2018b)	1a	Within	Trial	—	33		688.00	712.00	−0.21	[−0.7, 0.27]	—	—	—	—
	Ball & Bugg (2018b)	1b	Within	Trial	—	33		808.00	785.00	0.14	[−0.34, 0.63]	—	—	—	—
	Ball & Bugg (2018b)	2a	Within	Trial	—	34		726.00	748.00	−0.21	[−0.69, 0.27]	—	—	—	—
	Ball & Bugg (2018b)	2b	Within	Trial	—	34		684.00	712.00	−0.17	[−0.65, 0.31]	—	—	—	—
	Kuhlmann & Rummel (2014)	1	Between	Trial	—	27	30	1,280.00	1,158.00	0.54	[0.02, 1.08]	—	—	—	—
	Loft et al. (2011)	1	Between	Trial	—	48	48	555.00	549.00	0.02	[−0.38, 0.42]	—	—	—	—
	Lourenco et al. (2013)	1	Between	Trial	—	38	39	850.00	810.00	0.21	[−0.24, 0.66]	—	—	—	—
	Pinto et al. (2022)	1	Between	Trial	—	71	70	1,728.00	1,711.00	0.03	[−0.3, 0.37]	—	—	—	—
	Pinto et al. (2022)	2	Between	Trial	—	68	67	1,644.00	1,632.00	0.02	[−0.32, 0.36]	—	—	—	—
	Combined (all)	—		All	25	—	—	919.65	901.70	0.11 (0.06)	[−0.01, 0.22]	[−0.26, 0.48]	38.11	37.03%	0.03
	Blocked subgroup	—		Blocked	10	—	—	784.70	747.30	0.24	[0.11, 0.38]	[0.09 0.40]	7.52	0.00%	0
	Proximity subgroup	—		Proximity	4	—	—	1,496.75	1,507.25	0.08	[−0.41, 0.56]	[−1.60, 1.75]	16.6	81.93%	0.21
	Trial subgroup	—		Trial	11	—	—	971.82	961.82	0.01	[−0.11, 0.12]	[−0.12, 0.14]	8.02	0.00%	0

RT: response time; DV: dependent variable; S: specific condition; NS: nonspecific condition; CI: confidence interval; PI: prediction interval.

**Figure 6.**
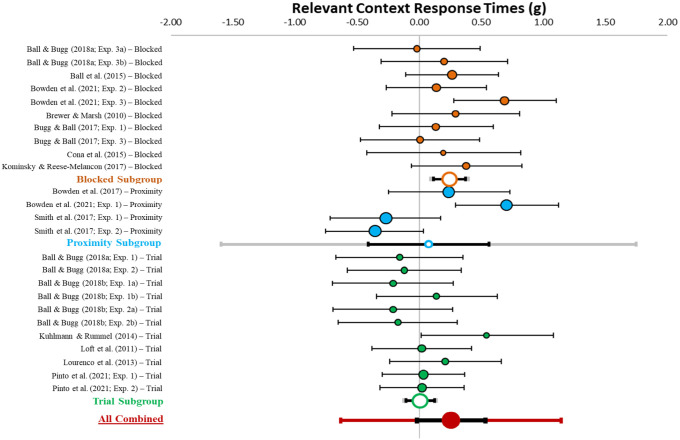
Forest plot for the effect of context specification on response times in the relevant context. *Note.* This figure shows the individual (coloured circles with black outlines), subgroup combined (white circles with coloured outlines), and overall combined (red circle) effect sizes for the effect of context specification on ongoing task response times in the relevant context with the relative weight of each data point on the left side. Black error bars reflect 95% confidence intervals and grey (subgroup) and red (overall combined) error bars reflect 95% prediction intervals.

The between group ANOVA indicated no significant specification differences in response times in relevant contexts between procedures (*p* = .054). As can be seen in Figure 6, for the subgroup analyses, the confidence interval did not overlap with zero for blocked procedure, but it did for the proximity and trial-level procedures. This suggests that anticipating targets in relevant contexts slows response times only for the blocked procedures. However, this should be interpreted with caution considering the nonsignificant between-procedure *p*-value.

#### Relevant context accuracy

**Figure 7.**
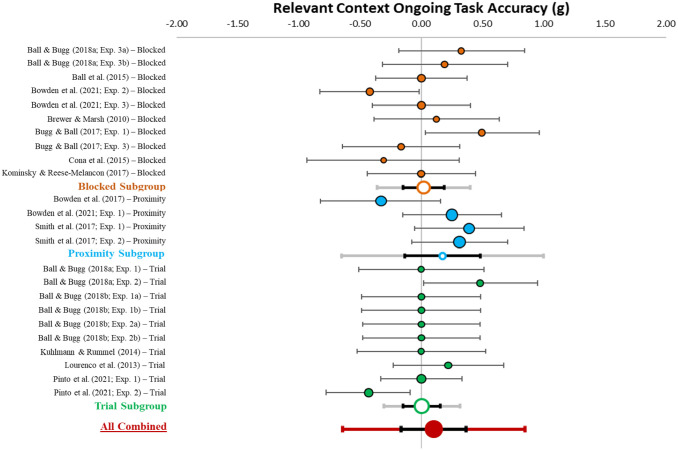
Forest plot for the effect of context specification on accuracy in the relevant context. *Note.* This figure shows the individual (coloured circles with black outlines), subgroup combined (white circles with coloured outlines), and overall combined (red circle) effect sizes for the effect of context specification on ongoing task accuracy in the relevant context with the relative weight of each data point on the left side. Black error bars reflect 95% confidence intervals and grey (subgroup) and red (overall combined) error bars reflect 95% prediction intervals.

Table 3 describes effect sizes and heterogeneity indices for the accuracy differences in relevant contexts between specific and nonspecific conditions, which are also displayed in Figure 7. The meta-analysis (*k* = 24) revealed a small-sized combined effect (*g* = 0.04, *z*-value = 0.78, *p* = .436, 95% CI [−0.07, 0.15], 95% PI [−0.27, 0.35]). Critically, the confidence and prediction intervals include zero. This suggests that having information about when a PM target will appear has no effect on ongoing task accuracy when the target is relevant. There was low heterogeneity among effect sizes (*Q* = 32.06, *p* = .099; *I*^2^ = 28.26; *T*^2^ = .02).

**Table 3.** List of studies, designs, means, effect sizes, confidence and prediction intervals, and heterogeneity indices for relevant context accuracy.

DV	Study	Exp	Design	Type	K	*N* (S)	*N* (NS)	Mean (S)	Mean (NS)	Hedge’s *g* (*SE*)	95% CI	95% PI	Q	I^2^	T^2^
Relevant ongoing task accuracy	Ball & Bugg (2018a)	3a	Between	Blocked	—	30	30	0.94	0.92	0.32	[−0.18, 0.84]	—	—	—	—
	Ball & Bugg (2018a)	3b	Within	Blocked	—	30		0.93	0.94	0.19	[−0.32, 0.7]	—	—	—	—
	Ball et al. (2015)	1	Between	Blocked	—	56	56	0.95	0.95	0	[−0.37, 0.37]	—	—	—	—
	Bowden et al. (2021)	2	Between	Blocked	—	48	48	0.92	0.89	−0.42	[−0.83, −0.02]	—	—	—	—
	Bowden et al. (2021)	3	Between	Blocked	—	48	48	0.89	0.89	0	[−0.4, 0.4]	—	—	—	—
	Brewer & Marsh (2010)	1	Between	Blocked	—	30	30	0.96	0.95	0.12	[−0.39, 0.63]	—	—	—	—
	Bugg & Ball (2017)	1	Between	Blocked	—	37	37	0.92	0.95	0.49	[0.03, 0.97]	—	—	—	—
	Bugg & Ball (2017)	3	Between	Blocked	—	35	33	0.93	0.92	−0.16	[−0.65, 0.31]	—	—	—	—
	Cona et al. (2015)	1	Between	Blocked	—	21	20	0.98	0.97	−0.31	[−0.94, 0.31]	—	—	—	—
	Kominsky & Reese-Melancon (2017)	1	Between	Blocked	—	40	40	0.96	0.96	0	[−0.44, 0.44]	—	—	—	—
	Bowden et al. (2017)	1	Between	Proximity	—	32	34	0.96	0.94	−0.02	[−0.82, 0.16]	—	—	—	—
	Bowden et al. (2021)	1	Between	Proximity	—	48	48	0.88	0.9	0.25	[−0.15, 0.65]	—	—	—	—
	R. E. Smith et al. (2017)	1	Between	Proximity	—	40	39	0.98	0.96	0.39	[−0.05, 0.84]	—	—	—	—
	R. E. Smith et al. (2017)	2	Between	Proximity	—	52	50	0.96	0.93	0.31	[−0.08, 0.71]	—	—	—	—
	Ball & Bugg (2018a)	1	Between	Trial	—	30	30	0.92	0.92	0	[−0.51, 0.51]	—	—	—	—
	Ball & Bugg (2018a)	2	Between	Trial	—	37	37	0.95	0.93	0.48	[0.02, 0.95]	—	—	—	—
	Ball & Bugg (2018b)	1a	Within	Trial	—	33		0.95	0.95	0	[−0.49, 0.49]	—	—	—	—
	Ball & Bugg (2018b)	1b	Within	Trial	—	33		0.93	0.93	0	[−0.49, 0.49]	—	—	—	—
	Ball & Bugg (2018b)	2a	Within	Trial	—	34		0.96	0.96	0	[−0.48, 0.48]	—	—	—	—
	Ball & Bugg (2018b)	2b	Within	Trial	—	34		0.94	0.94	0	[−0.48, 0.48]	—	—	—	—
	Kuhlmann and Rummel (2014)	1	Between	Trial	—	27	30	0.89	0.89	0	[−0.52, 0.52]	—	—	—	—
	Loft et al. (2011)	1	Between	Trial	—	48	48	—	—	—	—	—	—	—	—
	Lourenco et al. (2013)	1	Between	Trial	—	38	39	0.95	0.96	0.22	[−0.23, 0.67]	—	—	—	—
	Pinto et al. (2022)	1	Between	Trial	—	71	70	0.9	0.9	0	[−0.33, 0.33]	—	—	—	—
	Pinto et al. (2022)	2	Between	Trial	—	68	67	0.92	0.87	−0.43	[−0.78, −0.09]	—	—	—	—
	Combined (all)	—		All	24	—	—	0.94	0.93	0.04 (0.05)	[−0.07, 0.15]	[−0.27, 0.35]	32.06	28.26%	0.02
	Blocked subgroup	—		Blocked	10	—	—	0.94	0.93	0.02	[−0.15, 0.19]	[−0.36, 0.40]	12.52	28.13%	0.02
	Proximity subgroup	—		Proximity	4	—	—	0.95	0.93	0.17	[−0.13, 0.48]	[−0.65, 1.00]	5.73	47.64%	0.04
	Trial subgroup	—		Trial	10	—	—	0.93	0.93	0	[−0.15, 0.15]	[−0.31, 0.31]	11.37	20.86%	0.01

DV: dependent variable; S: specific condition; NS: nonspecific condition; CI: confidence interval; PI: prediction interval.

The between group ANOVA indicated no significant specification differences in accuracy in relevant contexts between procedures (*p* = .593). As can be seen in Figure 7, for the subgroup analyses the confidence interval overlapped with zero for all procedures, indicating that context specification does not influence ongoing task accuracy in any of the procedures.

#### Irrelevant context response times

**Figure 8.**
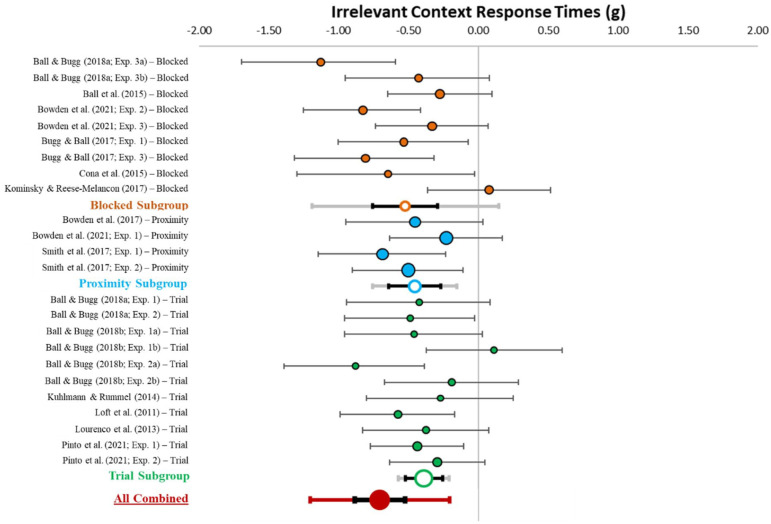
Forest plot for the effect of context specification on response times in the irrelevant context. *Note.* This figure shows the individual (coloured circles with black outlines), subgroup combined (white circles with coloured outlines), and overall combined (red circle) effect sizes for the effect of context specification on ongoing task response times in the irrelevant context with the relative weight of each data point on the left side. Black error bars reflect 95% confidence intervals and grey (subgroup) and red (overall combined) error bars reflect 95% prediction intervals.

Table 4 describes effect sizes and heterogeneity indices for the response time differences in irrelevant contexts between specific and nonspecific conditions, which are also displayed in Figure 8. The meta-analysis (*k* = 24) revealed a medium-sized combined effect (*g* = −0.45, *z*-value = −8.05, *p* < .001, 95% CI [−0.56, −0.33], 95% PI [−0.76, −0.13]). Critically, the confidence and prediction intervals do not include zero. This suggests that having information about when a PM target will appear speeds ongoing task response times when the target is irrelevant. There was low heterogeneity among effect sizes (*Q* = 32.17, *p* = .097; *I*^2^ = 28.52; *T*^2^ = .02).

**Table 4.** List of studies, designs, means, effect sizes, confidence and prediction intervals, and heterogeneity indices for irrelevant context RTs.

DV	Study	Exp	Design	Type	K	*N* (S)	*N* (NS)	Mean (S)	Mean (NS)	Hedge’s *g* (*SE*)	95% CI	95% PI	*Q*	*I* ^2^	*T* ^2^
Irrelevant ongoing task response time	Ball & Bugg (2018a)	3a	Between	Blocked	—	30	30	406	604	—1.13	[−1.69, −0.59]	—	—	—	—
	Ball & Bugg (2018a)	3b	Within	Blocked	—	30		683	742	−0.43	[−0.95, 0.08]	—	—	—	—
	Ball et al. (2015)	1	Between	Blocked	—	56	56	678	719	−0.27	[−0.65, 0.1]	—	—	—	—
	Bowden et al. (2021)	2	Between	Blocked	—	48	48	721	835	−0.83	[−1.25, −0.42]	—	—	—	—
	Bowden et al. (2021)	3	Between	Blocked	—	48	48	756	809	−0.33	[−0.74, 0.07]	—	—	—	—
	Brewer & Marsh (2010)	1	Between	Blocked	—	30	30	—	—	—	—	—	—	—	—
	Bugg & Ball (2017)	1	Between	Blocked	—	37	37	515	613	−0.53	[−1.01, −0.07]	—	—	—	—
	Bugg & Ball (2017)	3	Between	Blocked	—	35	33	525	649	−0.81	[−1.32, −0.32]	—	—	—	—
	Cona et al. (2015)	1	Between	Blocked	—	21	20	790	889	−0.65	[−1.3, −0.03]	—	—	—	—
	Kominsky & Reese-Melancon (2017)	1	Between	Blocked	—	40	40	681	672	0.08	[−0.36, 0.52]	—	—	—	—
	Bowden et al. (2017)	1	Between	Proximity	—	32	34	1,644.00 753.00	1,830	−0.45	[−0.95, 0.03]	—	—	—	—
	Bowden et al. (2021)	1	Between	Proximity	—	48	48	1,522	787	−0.23	[−0.64, 0.17]	—	—	—	—
	R. E. Smith et al. (2017)	1	Between	Proximity	—	40	39	1,222	1,927	−0.68	[−1.15, −0.23]	—	—	—	—
	R. E. Smith et al. (2017)	2	Between	Proximity	—	52	50	868	1,479	−0.5	[−0.90, −0.11]	—	—	—	—
	Ball & Bugg (2018a)	1	Between	Trial	—	30	30	845	918	−0.43	[−0.95, 0.09]	—	—	—	—
	Ball & Bugg (2018a)	2	Between	Trial	—	37	37	726	918	−0.49	[−0.96, −0.03]	—	—	—	—
	Ball & Bugg (2018b)	1a	Within	Trial	—	33		817	791	−0.46	[−0.96, 0.03]	—	—	—	—
	Ball & Bugg (2018b)	1b	Within	Trial	—	33		665	796	0.11	[−0.38, 0.6]	—	—	—	—
	Ball & Bugg (2018b)	2a	Within	Trial	—	34		707	757	−0.88	[−1.39, −0.39]	—	—	—	—
	Ball & Bugg (2018b)	2b	Within	Trial	—	34		1,103	743	−0.19	[−0.67, 0.29]	—	—	—	—
	Kuhlmann and Rummel (2014)	1	Between	Trial	—	27	30	389	1,161	−0.27	[−0.8, 0.25]	—	—	—	—
	Loft et al. (2011)	1	Between	Trial	—	48	48	749	538	−0.58	[−0.99, −0.17]	—	—	—	—
	Lourenco et al. (2013)	1	Between	Trial	—	38	39	1,482	810	−0.37	[−0.83, 0.08]	—	—	—	—
	Pinto et al. (2022)	1	Between	Trial	—	71	70	1,465	1,697	−0.44	[−0.78, −0.11]	—	—	—	—
	Pinto et al. (2022)	2	Between	Trial	—	68	67		1,610	−0.29	[−0.64, 0.05]	—	—	—	—
	Combined (all)	—	—	All	24	—	—	863	970.58	−0.45 (0.06)	[−0.56, −0.33]	[−0.76, −0.13]	32.17	28.52%	0.02
	Blocked subgroup	—	—	Blocked	9	—	—	639.44	725.78	−0.52	[−0.76, −0.29]	[−1.19, 0.15]	18.35	56.40%	0.07
	Proximity subgroup	—	—	Proximity	4	—	—	1,285.25	1,505.75	−0.46	[−0.64, −0.27]	[−0.76, −0.15]	2.28	0.00%	0
	Trial subgroup	—	—	Trial	11	—	—	892.36	976.27	−0.39	[−0.52, −0.26]	[−0.57, −0.17]	10.41	3.95%	0

RT: response time; DV: dependent variable; S: specific condition; NS: nonspecific condition; CI: confidence interval; PI: prediction interval.

The between group ANOVA indicated no significant specification differences in accuracy in irrelevant contexts between procedures (*p* = .601). As can be seen in Figure 8, for the subgroup analyses the confidence intervals do not overlap with zero for any of the procedures, indicating that context information can be used to reduce monitoring in irrelevant contexts regardless of procedure type.

#### Irrelevant context accuracy

**Figure 9.**
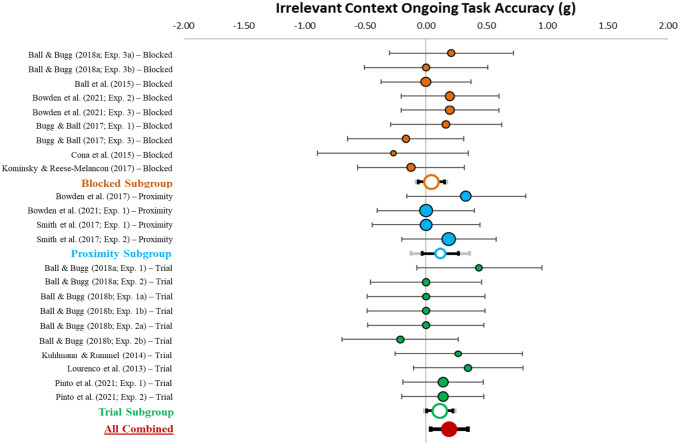
Forest plot for the effect of context specification on accuracy in the irrelevant context. *Note.* This figure shows the individual (coloured circles with black outlines), subgroup combined (white circles with coloured outlines), and overall combined (red circle) effect sizes for the effect of context specification on ongoing task accuracy in the irrelevant context with the relative weight of each data point on the left side. Black error bars reflect 95% confidence intervals and grey (subgroup) and red (overall combined) error bars reflect 95% prediction interval.

Table 5 describes effect sizes and heterogeneity indices for the accuracy differences in irrelevant contexts between specific and nonspecific conditions, which are also displayed in Figure 9. The meta-analysis (*k* = 23) revealed a small-sized combined effect (*g* = 0.09, *z* = 2.58, *p* = .010, 95% CI [0.02, 0.16], 95% PI [0.02, 0.16]). Critically, the confidence and prediction intervals do not include zero. This suggests that having information about when a PM target will appear improves ongoing task accuracy when the target is irrelevant. There was low heterogeneity among effect sizes (*Q* = 12.03, *p* = .957; *I*^2^ = 0.00; *T*^2^ = .00).

**Table 5.** List of studies, designs, means, effect sizes, confidence and prediction intervals, and heterogeneity indices for irrelevant context accuracy.

DV	Study	Exp	Design	Type	K	*N* (S)	*N* (NS)	Mean (S)	Mean (NS)	Hedge’s *g* (*SE*)	95% CI	95% PI	*Q*	*I* ^2^	*T* ^2^
															
Irrelevant ongoing task accuracy	Ball & Bugg (2018a)	3a	Between within	Blocked	—	30	30	0.97	0.96	0.21	[−0.3, 0.72]	—	—	—	—
	Ball & Bugg (2018a)	3b	Between	Blocked	—	30		0.94	0.94	0	[−0.51, 0.51]	—	—	—	—
	Ball et al. (2015)	1	Between	Blocked	—	56	56	0.97	0.97	0	[−0.37, 0.37]	—	—	—	—
	Bowden et al. (2021)	2	Between	Blocked	—	48	48	0.92	0.91	0.2	[−0.2, 0.6]	—	—	—	—
	Bowden et al. (2021)	3	Between	Blocked	—	48	48	0.92	0.91	0.2	[−0.2, 0.6]	—	—	—	—
	Brewer & Marsh (2010)	1	Between	Blocked	—	30	30	—	—	—	—	—	—	—	—
	Bugg & Ball (2017)	1	Between	Blocked	—	37	37	0.97	0.96	0.16	[−0.29, 0.63]	—	—	—	—
	Bugg & Ball (2017)	3	Between	Blocked	—	35	33	0.96	0.97	−0.16	[−0.65, 0.31]	—	—	—	—
	Cona et al. (2015)	1	Between	Blocked	—	21	20	0.96	0.97	−0.27	[−0.9, 0.35]	—	—	—	—
	Kominsky & Reese-Melancon (2017)	1	Between	Blocked	—	40	40	0.93	0.94	−0.12	[−0.57, 0.32]	—	—	—	—
	Bowden et al. (2017)	1	Between	Proximity	—	32	34	0.97	0.96	0.33	[−0.16, 0.82]	—	—	—	—
	Bowden et al. (2021)	1	Between	Proximity	—	48	48	0.9	0.9	0	[−0.4, 0.4]	—	—	—	—
	R. E. Smith et al. (2017)	1	Between	Proximity	—	40	39	0.96	0.96	0	[−0.44, 0.44]	—	—	—	—
	R. E. Smith et al. (2017)	2	Between	Proximity	—	52	50	0.94	0.92	0.19	[−0.16, 0.82]	—	—	—	—
	Ball & Bugg (2018a)	1	Between	Trial	—	30	30	0.95	0.93	0.44	[−0.07, 0.96]	—	—	—	—
	Ball & Bugg (2018a)	2	Within	Trial	—	37	37	0.92	0.92	0	[−0.46, 0.46]	—	—	—	—
	Ball & Bugg (2018b)	1a	Within	Trial	—	33		0.95	0.95	0	[−0.49, 0.49]	—	—	—	—
	Ball & Bugg (2018b)	1b	Within	Trial	—	33		0.95	0.95	0	[−0.49, 0.49]	—	—	—	—
	Ball & Bugg (2018b)	2a	Within	Trial	—	34		0.95	0.95	0	[−0.48, 0.48]	—	—	—	—
	Ball & Bugg (2018b)	2b	Between	Trial	—	34		0.95	0.96	−0.21	[−0.69, 0.27]	—	—	—	—
	Kuhlmann and Rummel (2014)	1	Between	Trial	—	27	30	0.92	0.9	0.27	[−0.26, 0.8]	—	—	—	—
	Loft et al. (2011)	1	Between	Trial	—	48	48	—	—	—	—	—	—	—	—
	Lourenco et al. (2013)	1	Between	Trial	—	38	39	0.95	0.93	0.35	[−0.1, 0.8]	—	—	—	—
	Pinto et al. (2022)	1	Between	Trial	—	71	70	0.91	0.9	0.14	[−0.19, 0.47]	—	—	—	—
	Pinto et al. (2022)	2		Trial	—	68	67	0.92	0.91	0.14	[−0.2, 0.48]	—	—	—	—
	Combined (all)	—		All	23	—	—	0.94	0.94	0.09 (0.03)	[0.02, 0.16]	[0.02, 0.16]	12.03	0.00%	0
	Blocked subgroup	—		Blocked	9	—	—	0.95	0.95	0.04	[−0.06, 0.15]	[−0.08, 0.17]	4.28	0.00%	0
	Proximity subgroup	—		Proximity	4	—	—	0.94	0.94	0.12	[−0.03, 0.27]	[−0.12, 0.36]	1.49	0.00%	0
	Trial subgroup	—		Trial	10	—	—	0.94	0.93	0.11	[0.0, 0.22]	[−0.01, 0.24]	5.72	0.00%	0

DV: dependent variable; S: specific condition; NS: nonspecific condition; CI: confidence interval; PI: prediction interval.

The between group ANOVA indicated no significant specification differences in accuracy in irrelevant contexts between procedures (*p* = .761). As can be seen in Figure 9, for the subgroup analyses, the confidence interval overlapped with or included zero for all procedures, indicating that context specification does not significantly influence ongoing task accuracy in any of the individual procedures alone. Note that this differs from the overall combined effect (with greater power) that shows a small benefit to accuracy from context.

### Secondary analysis summary

At the meta-analytic level, context specification affected both response times and accuracy. Specification had no overall effect on response times in relevant contexts and sped response times in irrelevant contexts (medium effect). For accuracy, specification had no effect on accuracy in relevant contexts but improved accuracy in irrelevant contexts (small effect). The effect of specification on response times in the relevant context differed by subgroup, with small to medium-sized effect observed in the blocked procedure and a nonsignificant effect observed in the proximity and trial-level procedure. Heterogeneity in effect sizes was low for response times and accuracy in both relevant and irrelevant contexts.

## Discussion

The present meta-analysis aimed to quantify overall effects of context specification on traditional metrics of strategic monitoring. Overall, we found support for our three primary hypotheses: (a) context specification improves PM performance, (b) the magnitude of these effects depends on the procedure, and (c) the specification benefit to PM is largely related to increases in monitoring in relevant contexts rather than decreases in monitoring in irrelevant contexts. Of secondary interest, we also found that context specification speeds ongoing task response times and increases accuracy overall when the PM target is irrelevant. These findings suggest that specification of when targets will occur when forming intentions not only improves PM performance, but also frees attentional resources by reducing monitoring demands in irrelevant contexts. However, this interpretation is qualified by the type of procedure used, such that having a context that varies randomly will not improve the likelihood of realising an intention. These results have important theoretical implications and lead to practical recommendations for researchers discussed below.

Strategic monitoring allows an individual to flexibly allocate attention towards different goals depending on the context in which they find themselves (e.g., Marsh et al., 2006). When the PM target is relevant, target checking can occur, but doing so can come at a cost to ongoing task performance. When the context is not relevant, one can disengage monitoring and direct attention towards the ongoing task to reduce cost. Moderator analyses suggest that people who use context information to slow ongoing task responses in the relevant context detect more PM targets. Critically, however, people who only used context information to conserve attentional resources by monitoring less when the context was irrelevant did not detect more PM targets. Importantly, these results show that conserved attentional resources are not necessarily reallocated effectively, but effective reallocation (i.e., more monitoring in the relevant context) predicts whether specification improves PM performance.

Strategic monitoring is accomplished by forming an intention to complete a future action in a specified context, identifying the context, and then using the outcome of the context decision to either engage or disengage target checking. Context identification is a critical process in strategic monitoring (Ball & Bugg, 2018b; Kuhlmann & Rummel, 2014) that dictates how attention is allocated dynamically depending on the context. However, identifying the context can be demanding, as it adds another cognitive operation that must be completed. The frequency of context identification determines the cognitive demand it places on the individual and is likely easier to accomplish in some procedures than others. In the blocked and proximity procedures, participants only need to identify the context on or before the first trial of a context change and then use that information to engage or disengage target checking on subsequent trials. Conversely, the context is unpredictable in the trial-level procedure and participants must identify the context and apply the appropriate process (i.e., engage or disengage target checking) on every single trial.

The dual mechanisms of control framework (Braver et al., 2007) posits that cognitive control is exerted through proactive and reactive control mechanisms. Proactive control sustains goal activation (e.g., context identification and target checking) that biases attention towards goal-relevant stimuli prior to presentation. In contrast, reactive control relies on bottom-up or stimulus-driven activation of goal-relevant representations. Bugg et al. (2013) argue that proactive control is necessary for sustained monitoring and facilitates realising an intention through consistent target checking, but this comes at a cost to ongoing task performance. While proactive target checking (i.e., monitoring) is most effective at realising PM intentions, it is cognitively demanding, difficult to sustain, and wanes in consistency (i.e., effectiveness) over long time periods (West et al., 2002). Advanced knowledge about when PM targets occur can facilitate proactive context identification (and subsequently the engaging and disengaging of target checking) and reduce the amount of time it needs to be sustained, ultimately benefitting PM performance. Specification in the blocked and proximity procedures allows one to conserve cognitive resources and exert more effective proactive context identification in the relevant context, while those without context information must utilise cognitive resources to target check over the entire duration and are more likely to experience lapses in target checking that lead to target misses. Because the trial-level procedure is unpredictable, context identification and target checking can only occur reactively (Ball & Bugg, 2018a). While costs are reduced (and resources conserved) in the irrelevant context of the trial-level procedure, having to rely on reactive control to engage target checking may offset the benefits of conserving cognitive resources and lead to no differences in PM performance between those with and without specification.

An alternative interpretation that extends the context identification explanation involves the role of task switching (Rogers & Monsell, 1995). A typical task-switching paradigm involves performing two separate task decisions (e.g., lexical decisions and syllable counting) that use the same stimuli (e.g., words and nonwords) with a cue that specifies which task to perform (e.g., red font = lexical decisions; green font = syllable counting). In the switching literature, a *task-set* refers to the instructions (e.g., press the F or J key) and goals (e.g., identify a stimulus as a word or nonword) for a single task (e.g., lexical decisions). Task-set reconfiguration occurs when the same stimulus requires a different goal and/or instructions (e.g., count syllables), and cognitive control is needed to reconfigure a task-set. A cost to performance (i.e., response times and/or accuracy) is observed on *switch trials* when the task-set must be reconfigured compared with when the same task-set is performed successively on *repetition trials* (Jersild, 1927). Important for this study is the differentiation between endogenous and exogenous control. Endogenous control is exerted in advance of stimulus onset, whereas exogenous control is exerted in response to the stimulus. Critically, when task-set reconfiguration can be anticipated (endogenous control), either explicitly with a pretrial context cue or implicitly with a predictable pattern of switch-trials, switching costs are reduced compared with when task-set reconfiguration cannot be anticipated (exogenous control; Rogers & Monsell, 1995).

The cue in a task-switching procedure is similar to the context information (e.g., colour, location, word type) in a strategic monitoring paradigm that signals whether a target check should be made. That is, context serves as a switch cue that tells a participant whether they should adopt the ongoing task task-set (i.e., focus on the ongoing task alone) or the PM task-set (i.e., target check and complete the ongoing task). Context switch trials require goal updating, and the previous goal may interfere with the present goal in a way that interferes with processing on that trial. It is therefore possible that switching from irrelevant to relevant contexts on a target trial can interfere with target checking. Support for this interpretation comes from the study by Bowden et al. (2021) that examined PM performance for targets that appeared on context switch trials (i.e., first trial of the relevant context) and compared groups that had a pretrial context cue to groups that did not. Critically, groups that had a pretrial context cue were able to identify the relevant context prior to the target stimulus onset. Results from two out of their three experiments showed that only with a pretrial context cue can context specification improve PM performance when the target appears on a context switch trial. In the blocked and proximity procedures, the switch cue of context can be anticipated, either implicitly or explicitly, and then a single task-set maintained across multiple (i.e., repetition) trials. In contrast, the trial-level procedure has a greater cognitive demand due to an unpredictable switch cue and switch trials occurring randomly on each trial. Our results support this interpretation in that context specification benefits PM performance in the blocked and proximity procedure, but not the trial-level procedure. Thus, for context to be beneficial to PM target detection, context identification demands must be minimised by making context predictable and reducing the number of times it must be identified.

Finally, it is worth noting the similarities between strategic monitoring in the event-based PM studies described herein and clock checking behaviour in time-based PM studies. In a typical time-based PM study, participants are given a specific time to make a PM response (e.g., every 3 min) and participants can press a button to display a clock with the current elapsed time. Clock checking behaviour often shows an “j”-shaped function, where participants initially check the clock to see the current time (e.g., 10 s), decrease checking during intermediate intervals, and then increase checking as the target PM interval approaches (e.g., 2 min 50 s). The frequency of clock checking in the interval immediately before the target time (e.g., 2:50–2:59) is an index of strategic “monitoring” and is associated with better PM performance (Jager & Kliegel, 2008; Joly-Burra et al., 2022; McFarland & Glisky, 2009; Mioni et al., 2020; Mioni & Stablum, 2014). Strategic clock checking behaviour may be like strategic monitoring in the event-based proximity procedure, where participants use spatial information (e.g., trial counters) to slow ongoing task responding in the relevant contexts (e.g., trials 25-30) that improves PM performance. One primary difference between the two task types is that context features in the event-based proximity procedure are externally cued (i.e., by the trial counter), whereas in a time-based task temporal information is maintained internally (i.e., a mental clock counter). Interestingly, participants in the event-based blocked procedure may also maintain an internal trial counter, whereby participants learn to predict the change in contexts every few (e.g., 8) trials. Indeed, prior research has shown that strategic monitoring in irrelevant contexts shows a “u”-shaped function (Ball et al., 2020; Lourenco & Maylor, 2014), where participants show slowing in the trials immediately after the context change (e.g., trials 1-3), faster responding in the intermediate trials (e.g., 4-8), and then slower responding near the anticipated context change (e.g., trials 9-10). In contrast to the blocked and proximity procedures, there are no spatiotemporal contextual cues to facilitate monitoring in the trial-level procedure. Future research directly comparing event-based and time-based PM may provide a clearer mechanistic account of how strategic monitoring facilitates PM performance (Marsh et al., 2006).

Although the results of the current meta-analysis are fairly straightforward, there are still a few remaining issues. First, the subgroup analysis did not completely account for the moderate heterogeneity among effect sizes in PM performance. The heterogeneity was still moderate in the blocked and proximity procedures, suggesting a wide range of true effect sizes in the population not due to procedure alone. However, the moderator analysis using relevant context ongoing task response times clearly predicted the effect of context specification on PM performance, suggesting this may be a way to better account for the entire heterogeneity in PM performance. It is also possible that other task features such as context type (e.g., colour, location, and word type) or ongoing task difficulty also account for heterogeneity in PM performance. Second, it should be noted that there was a relatively small sample (*k* = 4) of experiments using the proximity procedure. While this reaches the recommended minimum number of studies required for subgroup analyses (Fu et al., 2011), it does highlight that more research is needed using this procedural variant. A third important point to consider is whether context specification influences the perceived importance of the PM task, because previous research has shown stressing the importance of the PM task improves performance (Walter & Meier, 2014). Future strategic monitoring research would do well to assess perceived importance of the PM task in a postexperimental questionnaire to rule out this possibility. Finally, although we reasoned procedures differ in the demands placed on context identification, there is no direct measure of context identification, and no previous work has examined this explicitly. Future studies should directly compare context identification in blocked, proximity, and trial-level procedures in the same experiment. Examining procedural differences in a highly controlled way would enable the researcher to compare the demands of an unpredictable context and constantly varying task goals in the trial-level procedure with the more predictable context and consistent task goals in the blocked and proximity procedures. We would expect longer response times on context switch trials in the trial-level procedure compared with context switch trials in the blocked and proximity procedures, as well as PM performance results consistent with those observed in the present meta-analysis.

This study was partly motivated by previous research showing that laboratory-based PM tasks do not always correlate with real-world PM (Unsworth et al., 2012). One main difference between the two settings is that in naturalistic studies, participants are able to use a host of cognitive processes that may benefit PM performance that are typically controlled for in laboratory settings, including the ability to use context information to allocate attention towards fulfilling an intention. For example, many everyday PM intentions occur in a familiar environment. A person familiar with the location of a pharmacy (specific) that serves as the target to retrieve the intention to pick up a medication likely benefits from the spatiotemporal context information. It is therefore possible that providing predictable context information in laboratory PM tasks (i.e., specific condition) may be a more ecologically valid and better predictor naturalistic PM.

The current meta-analysis suggests context information can be used to flexibly allocate attention to realise future intentions while conserving cognitive resources when the context is irrelevant. These results can provide guidance for researchers studying strategic monitoring in the future, such that specific procedures can be recommended depending on the research question. For example, it would be prudent to employ a proximity or blocked procedure for research questions focusing on PM performance or relevant context slowing, while a trial-level procedure would suffice when looking at resource conservation in the irrelevant context. Outside of the laboratory, someone looking for a pharmacy may slow down or pay less attention to the road and cars around them. By knowing the relative location of the pharmacy, one can minimise these costs until the grocery store is nearby while also increasing intention fulfilment. Considering the demands of daily life and managing multiple intentions (e.g., attending meetings, sending critical emails, remembering appointments, and picking up children from school after work), carefully encoding the *retrieval* context for each intention can reduce the impact of maintaining an intention on one’s current activity (e.g., writing a grant or manuscript) while enhancing the likelihood of remembering to complete an intention.

## Notes

1 An additional rational for not including time-based PM strategic monitoring studies was because the contextual properties in time-based PM operate differently from context in event-based PM (i.e., temporal versus spatiotemporal), and strategic monitoring in time-based PM is measured by clock checking behaviour as the intention approaches. Thus, the metrics of strategic monitoring differ too much to compare time-based and event-based PM in the same analysis.
